# William Blair

**Published:** 1823-01

**Authors:** 


Died, early last month, at his house in Great Russell-street, William Blair, Esq. a gentleman of considerable eminence in his profession. Mr. Blair was born of Scotch parents in Suffolk, and was educated under Dr. Grimwood at Colchester. He came to London about the age of twenty-two, and was fixed with Mr. Pearson as a house-pupil. He became house-surgeon to the Lock Hospital in 1790, and was elected one of the surgeons to that charity in 1794. This situation he resigned in the year 1819. He was also surgeon, first to the Finsbury, and subsequently to the Bloomsbury Dispensary, and to the Rupture Society. Mr. Blair had been declining in health for upwards of two years: the symptoms which he laboured under indicated some disease of the heart; the exact nature of which, however, could not be known, as his body was not permitted to be examined after his death. Mr. Blair was the author of a small work on the use of the Nitrous Acid in Lues Venerea, and of a few papers in the various Medical Journals.

